# Cognitive dysfunction in Moyamoya disease: latest developments and future directions

**DOI:** 10.3389/fnhum.2024.1502318

**Published:** 2024-12-06

**Authors:** Xilong Wang, Ziqi Liu, Zhenyu Zhou, Junze Zhang, Yanru Wang, Shihao He, Rong Wang

**Affiliations:** ^1^Department of Neurosurgery, Beijing Tiantan Hospital, Capital Medical University, Beijing, China; ^2^Department of Pathology, Stanford University School of Medicine, Stanford, CA, United States; ^3^Department of Neurosurgery, Peking Union Medical College Hospital, Chinese Academy of Medical Sciences, Beijing, China; ^4^China National Clinical Research Center for Neurological Diseases, Beijing, China

**Keywords:** cognitive function, Moyamoya disease, neuropsychology, cognitive impairment, revascularization

## Abstract

Cognitive dysfunction is common in Moyamoya disease (MMD). However, current knowledge of cognitive impairment in MMD is inadequate. In this review, we explored the characteristics of altered cognitive function associated with MMD and offered recommendations aimed at guiding potential research endeavors into the cognitive dysfunction in MMD. Cognitive functions, including executive function, intelligence, memory and so on, show characteristic declines in MMD. The effects of cerebral revascularization surgery on cognitive impairment are controversial. Currently, there is still a lack of relevant research on cognitive impairment. Research on the pathogenesis and etiology associated with Moyamoya disease as well as long-term cohort studies, are important future directions.

## Introduction

In 1969, a specific cerebrovascular disorder was first identified as moyamoya disease (MMD), characterized by the progressive narrowing or even occlusion of the end of internal carotid arteries and the initiation of middle cerebral arteries and by the abnormal angiogenesis and abnormal vessel-network formation at the base of the skull (Suzuki and Takaku, 1969). MMD can result in complicated cerebral hemodynamic changes, causing severe ischemic and hemorrhagic accidents, which demonstrates two peak episodes in the first and the fourth decades of life (Scott and Smith, 2009; Kuroda and Houkin, 2008). However, in the clinical settings, MMD patients present in a variety of manifestations (Research Committee on the Pathology et al., 2012). And a certain percentage of patients suffer primarily from cognitive impairment, which greatly impacts their daily existence and overall well-being (Weinberg et al., 2011; Kronenburg et al., 2018; Antonov et al., 2023). In addition, despite decades of increasing understanding of MMD, its etiology and pathogenesis are still unclear (Gonzalez et al., 2023; Antonov et al., 2023). There is still a lack of approaches to evaluate the occurrence and progression of MMD, and currently, interventions for MMD continue to focus on surgical revascularizations to ameliorate or replace the patients’ intracranial hemodynamic abnormalities (Ihara et al., 2022; Gonzalez et al., 2023). In the context, cognitive function changes in MMD remains greatly under-recognized.

In this review, we searched the publications limited to the English language in Pubmed database using the keywords “cognition” and “moayamoya disease” with a cutoff date of September 1, 2024, and the writing group of five neurosurgeons read the results in detail, discussed several times and summarized the related findings. We explored the characteristics of altered cognitive function associated with MMD through a comprehensive study of the natural history, evaluation methods and prognosis of reported studies related to cognitive changes in MMD. Furthermore, we offered recommendations aimed at guiding potential research endeavors into the domination of cognitive function in relation to MMD.

## Natural history and cognitive impairment in MMD

The natural history of MMD is variable and elusive and the conditions in the majority of patients will progress gradually, regardless of the course and clinical manifestations (Scott and Smith, 2009). A retrospective study has shown that in pediatric MMD patients presenting with transient ischemic attacks (TIAs), 91.7% (*n* = 55) had recurrences of TIAs and 23.3% (*n* = 14) had subsequent strokes (Zhao et al., 2017). In a study with an average follow-up of more than 82 months of a Korean cohort including 241 hemodynamically stable adult MMD patients, the annual stroke risk was 4.5% (Cho et al., 2015). Another study with median 10.1 years follow-up in patients with hemorrhagic stroke reported that the annual incidence of recurrent hemorrhages averaged 4.5%, and the cumulative risk of rebleeding amounted to 7.8% after 5 years, escalated to 22.6% by the 10-year mark, and further increased to 35.9% by the 15-year point (Kang et al., 2019). A comparative analysis conducted within a single, multiethnic cohort of 250 hemispheres indicated that MMD in Asians may exhibit a more aggressive progression (*p* = 0.45) over an average follow-up period of 3.3 years (Feghali et al., 2019). Furthermore, a study indicated that asymptomatic MMD patients experienced an annual radiographic infarction or hemorrhage rate of 1.7% (Lai et al., 2022). In conclusion, MMD is a chronic progressive disease that can occur in children or adults with cerebral ischemia or cerebral hemorrhage, or even severe strokes, as the primary clinical manifestation.

With the advancement of the demands for quality of life, the cognitive impairment in MMD is receiving increasing attention. However, there are few studies related to the natural history of cognitive impairment associated with MMD.

Cognitive impairments in MMD are widespread. In a long-term cognitive function follow-up of sixty-one adult patients with MMD, it was found that cognitive dysfunction was very common in adult MMD with executive functions being the most commonly affected domain, followed by Performance IQ, processing speed, and visual memory at baseline (Chan et al., 2023). In a study comparing forty-nine adult MMD patients with twenty-three healthy controls, the cognitive impairment in MMD in some domains was identified with the *p*-values less than 0.05, including intelligence quotient, prospective memory, verbal fluency, attention, retrospective memory, Stroop Test, Continuous Performance Test, and Trail-Making Test Part A (Shi et al., 2020). Subsequently, another recent independent study showed that compared to healthy controls, adult MMD patients without stroke had significantly worse performance in Montreal Cognitive Assessment, Trail Making Test Part A, Trail Making Test Part B, clock drawing test, and Chinese auditory verbal learning test which have been used to examine cognitive profiles in the areas of global cognition, speed of information processing, executive function, visuospatial function, memory and verbal memory, respectively (Shen X. X. et al., 2023). In summation, cognitive dysfunction is common in adult MMD and cognitive impairments are mainly manifested in specific domains such as executive function, processing speed, intelligence quotient, visual and verbal memory.

Currently, the studies on the characteristics of cognitive impairment in children with MMD remain limited. In a prospective cohort study, fifteen of twenty-one children with MMD showed varying degrees of cognitive impairment (Li et al., 2019). In another recent study of cognitive impairment in pediatric MMD, the abnormal Frontal hemodynamics and the executive function deficits were recognized, regardless of stroke history and comorbidity (Choi et al., 2022). In another independent prospective cohort, patients with MMD typically exhibited cognitive test results that were below the average standard on cognitive function tests, and compared to adult patients, children performed better on processing speed and worse on visuospatial function (Kronenburg et al., 2023). And in a single-center retrospective study, 83 children with MMD had lower overall intelligence, executive function, and adaptability than average age-based measures (Gatti et al., 2023). Although current data are limited, these results indicate that pediatric MMD patients have also suffered from cognitive impairment, and its characteristics are different from those of adults. In children, the intelligence quotient and executive function are commonly affected domains, while in adults, impaired executive function is most common.

In summary, both adult and pediatric MMD patients generally suffer from cognitive dysfunction, and the characteristics of cognitive impairment of them are different. Further research and high-level evidence are needed to elucidate the cognitive impairment features of MMD.

Natural history of cognitive function in MMD remains unclear. During the baseline assessment and the three subsequent follow up visits (median = 2.31, 4.87, and 7.12 years), Chan and his colleagues found that there was no significant change in participants’ cognitive function assessment results and that their cognitive impairment was not associated with the age of onset, history of previous revascularization surgery, or history of previous stroke (Chan et al., 2023). Among sixty-eight adult patients with ischemic MMD without cerebral misery perfusion, there was no reduction in neuropsychological assessment outcomes during the 5-year follow-up period (Kitakami et al., 2022). In a 2-year prospective cohort study, the cognitive outcomes of seventy adult patients with ischemic MMD without cerebral misery perfusion also showed no significant change (Miyoshi et al., 2019). However, a previous study reported that in a follow-up of 26 unoperated adult hemorrhagic MMD patients and two control groups, Montreal Cognitive Assessment (MoCA) scores in the MMD group declined significantly year over year, especially in domains of delayed recall, visual space and executive function (Su et al., 2013). In a 5-year follow-up, deterioration or improvement in specific neuropsychologic tests was observed among adult MMD patients with different manifestations of cerebral microbleeds on magnetic resonance imaging (MRI) (Yabuki et al., 2022). In another 5-year follow-up study of cognitive function in twenty consecutive adults with MMD in Japan, the final Wilcoxon signed-rank test results showed a significant decrease in mental flexibility, programming, and inhibitory control than the results of the baseline evaluation (Nakamizo et al., 2022). In addition, the posterior cerebral artery scores in magnetic resonance angiography (MRA) steno-occlusive score system were significantly correlated with changes in cognitive assessment scores in this study (Nakamizo et al., 2022). The sample size, follow-up time, and clinical phenotypes of the participants may have contributed to the discrepancy between the results of these studies. The inconsistency of these results suggests that cognitive dysfunction associated with MMD might be the result of a combination of a variety of factors. In conclusion, there is still a lack of the studies of the natural history of cognitive dysfunction in MMD and further research is needed on the risk factors for the progression of cognitive impairment in MMD.

A recent study has conducted the systematic review and meta-analysis indicated that cognitive impairment linked to MMD is not correlated with a history of ischemic or hemorrhagic stroke (Toh et al., 2024). However, the previous study has reported greater impairment of cognitive function in MMD patients with hemorrhagic stroke compared with healthy and spontaneous intracerebral hemorrhage controls (Su et al., 2013). In a recent retrospective study involving consecutive 102 adult European patients with MMD, it was observed that around 33% (34 of 102) participants showed cognitive impairment related to cerebrovascular issues, with executive function being the most affected, succeeded by attention and processing speed (Giroud et al., 2024a). In addition, the further analysis of these results showed that a previous diagnosis of stroke, including ischemic stroke, was significantly associated with the domains of attention and processing speed, while executive function was associated with chronic ischemic lesions on MRI (Giroud et al., 2024a). Despite the negative conclusion of the recent meta-analysis, further evidence is needed to confirm the correlation between stroke and cognitive impairment. The other results suggest that stroke might play a role in MMD-related cognitive impairment.

In addition, in our previous study, we observed that twenty-one asymptomatic adult MMD patients exhibited a decline in cognitive performance in certain domains compared with twenty normal controls, including intelligence, spatial imagination, working memory, and computational ability (He et al., 2020). These results suggested that cognitive impairment was not only caused by previous stroke, but might also be influenced by long-term hypoperfusion.

In conclusion, although the studies of natural history of cognitive function related to MMD is insufficient, we could preliminarily conclude from the above data that MMD patients may exhibit progressive cognitive dysfunction in specific domains, and that cerebral hemorrhage and hemodynamic impairment may be closely related to it.

## Neuroimaging findings and mechanisms of cognitive dysfunction in MMD

For many years, considerable effort has been dedicated to correlating cognitive impairment with objective imaging findings in MMD, in order to elucidate the mechanisms underlying cognitive dysfunction associated with MMD.

In a neuroradiological study, it was reported that cognitive impairment associated with adult MMD was found to be strongly associated with cerebral cortical infarction, and that there appeared to be a potential positive correlation between the CBF and cognitive function (Mogensen et al., 2012). However, in another study of adult MMD, it was found that the cognitive dysfunction in several domains such as executive function could occur even when brain scans were completely normal (Karzmark et al., 2012). Subsequently, a number of studies have shown that CBF deficiency is strongly associated with cognitive dysfunction in both adult and pediatric MMD (Kang et al., 2017; Kazumata et al., 2020; Shen X. X. et al., 2023; Zou et al., 2023). Shen and his colleagues established a prospective cohort of 115 stroke-free adult MMD patients and 82 healthy controls who underwent cognitive tests and dynamic susceptibility contrast-enhanced (DSC) MRI (Shen X. X. et al., 2023). The results shows that the CBF in the bilateral lateral frontal lobes, centrum semiovale, and temporal lobes was significantly correlated to the score of executive function, and the CBF in the left centrum semiovale and temporal lobes was significantly correlated to the scores of processing speed and verbal memory (Shen X. X. et al., 2023). And a recent study revealed that MRI could be used to assess CBF in MMD and that CBF reduction could be identified as an independent factor of cognitive dysfunction in MMD (Giroud et al., 2024b). The application of MRI to evaluate cerebral perfusion provides a convenient, fast and non-invasive method for neurosurgeons to evaluate cognitive impairment in moyamoya disease.

In addition, previous studies found, by measuring cerebrovascular reserve (CVR) and apparent diffusion coefficient (ADC), that ADC elevation in frontal white matter of MMD adults correlates with reduced CVR and executive dysfunction, indicating its potential as a cognitive dysfunction marker (Calviere et al., 2012; Nakamizo et al., 2014).

As neuroimaging technology improves, an increasing number of examination modalities are being used to explore the relationship between brain microstructure and cognitive dysfunction, and the results of a number of studies have shown that disruptions in white matter microstructure and brain connectivity are strongly associated with cognitive impairment in MMD (Kazumata et al., 2015; Kazumata et al., 2017; Hara et al., 2018; Hara et al., 2019; Tsunoda et al., 2023). Hara and his colleagues built a cohort of 31 patients with MMD and 20 matched healthy controls, and applied neurite orientation dispersion and density imaging (NODDI) and ^15^O-gas positron emission tomography (PET) to them (Hara et al., 2019). The results suggested that the microstructure parameters obtained by NODDI were closely correlated to the metabolic and hemodynamic parameters obtained by PET, and were also closely correlated to the neurocognitive performance of patients with MMD (Hara et al., 2019). The results showed that PET can assess the hemodynamics of patients with MMD and is closely correlated to the microstructure of brain tissue which might be the pathological basis of neurocognitive disorders.

In a previous study, the diffusion tensor imaging (DTI) data obtained from MRI were utilized to uncover the mechanisms behind cognitive impairment (Liu et al., 2020). The results suggested that multiple parameters obtained from DTI, including fractional anisotropy and mean diffusivity, were significantly different in specific brain regions between the MMD and control groups (Liu et al., 2020). The mapping of these parameters of DTI showed that the cognitive impairment in ischemic MMD might be associated with white matter impairment (Liu et al., 2020). h. A study investigating the correlation between white matter hyperintensities (WMH) and cognitive dysfunction in asymptomatic adults with MMD indicated that a greater WMH load was strongly linked to overall cognitive deficits (Shen J. et al., 2023). Additionally, the extent of WMH was found to be significantly correlated with various aspects of cognitive function, such as memory and executive function (Shen J. et al., 2023). The findings indicate a strong correlation between the burden of WMH and multiple cognitive functions in individuals with asymptomatic MMD (Shen J. et al., 2023).

More interestingly, a study using resting-state functional MRI to evaluate the spatial patterns of brain activity in MMD suggested that the cognitive impairment was strongly associated with the specific low-frequency fluctuations (ALFF) performance patterns in MMD (Lei et al., 2014). Then, Lei and his colleagues built an adult cohort of 36 MMD patients with neurocognitive impairment, 43 without neurocognitive impairment and 26 healthy controls who all underwent resting-state functional MRI to calculate the functional connectivity and construct a sparse representation-based classifier (Lei et al., 2020). The results show that the cognitively impaired group had the lowest scores and the healthy controls had the highest scores, and the classifiers had high AUC, accuracy, sensitivity, and specificity for distinguishing MMD from healthy individuals. In addition, in a pediatric MMD cohort of 30 children, the hypercapnic challenge blood oxygen level-dependent functional MRI was performed to evaluate CVR, and the results shows that all children demonstrated frontal hemodynamic disorders and the compensation from the parietal and temporal lobes might play a role in alleviating cognitive impairment (Choi et al., 2022). The functional MRI fully utilizes its convenience, affordability, and accessibility in the assessment of the brain tissue in patients with MMD.

The above results suggest that cognitive dysfunction in MMD might be associated with lesions in the cerebral white matter, CBF, brain connectivity and so on. MRI and other approaches could be utilized to evaluate the status and predict prognosis of cognitive function in MMD.

## Treatment and prognosis of cognitive impairment in MMD

Currently, the interventions for MMD focus on surgical revascularizations to ameliorate or replace the patients’ intracranial hemodynamic abnormalities (Ihara et al., 2022). The influences of surgery on the prognosis of cognitive function related to MMD has been of great interest.

The previous studies have shown that revascularization surgery could increase localized CBF and improve cognitive dysfunction in MMD (Sato et al., 1990; Baek et al., 2014; Weinberg et al., 2011; Joshi et al., 2024; Kimura et al., 2022; Hsu et al., 2020; Yanagihara et al., 2019; Kazumata et al., 2019; Kim et al., 2018).

In a prospective cohort followed for 5 years, about one-third of patients with MMD showed no significant difference in cognitive function, one-third improved, and one-third deteriorated before and after surgical revascularization (Uchida et al., 2021). Among them, the former two subgroups had a significant increase in CBF before and after surgery, and the last one had a significant decrease in CBF (Uchida et al., 2021). In addition, there were no significant differences in cognitive test results at 2 months and 5 years after surgery in this cohort (Uchida et al., 2021). The results showed that cognitive outcomes might be worse in patients after revascularization surgery with poor CBF improvement (Uchida et al., 2021).

In a 3-year follow-up study of patients with MMD in their twenties, the authors collected 12 participants during 12 years, it was found that younger patients (>20 years) were more prone to hemodynamic disturbances and complications of cerebral hyperperfusion after combined revascularization compared to older patients (30–60 years), which might lead to worsening of cognitive dysfunction (Ono et al., 2024). An additional independent 5-year cohort study of 31 MMD patients also showed that there were 11 (35%) participants developing cerebral atrophy after revascularizations, and the results of SPECT suggested that cerebral atrophy was independently associated with cerebral hyperperfusion and cerebral hyperperfusion syndrome, and cerebral atrophy was associated with cognitive dysfunction which investigated that cerebral hyperperfusion following revascularization for MMD might result in brain atrophy among adult patients, leading to persistent cognitive dysfunction (Katakura et al., 2022).

Moreover, the majority of patients in an 84-patient study of revascularization surgery for adult MMD showed little change in cognitive function postoperatively compared with preoperatively (Zeifert et al., 2017). These recent findings seem to make us aware of the limited short-term effect of revascularization on cognitive impairment in adult patients with MMD. And these findings suggest to us in addition that cognitive dysfunction in MMD may be the result of a combination of CBF and other factors. The multi-center, multi-ethnic, long-term follow-up studies are urgently needed.

There is still a lack of specific drugs for MMD management. However, antiplatelet drugs and lipid-lowering medications are promising strategies to slow MMD progression (Ihara et al., 2022; Research Committee on the Pathology et al., 2012; Ando et al., 2019). In a prospective cohort study with over 2 years of follow-up, cilostazol demonstrated a greater improvement in cognitive impairment among adults with MMD compared to clopidogrel (Ando et al., 2019). The results suggest that cognitive impairment in MMD may be alleviated by medication.

Despite the lack of specific treatment strategies, cognitive dysfunction in MMD can still benefit from revascularization and medication. Therefore, there is an increasing emphasis on the development of appropriate treatment strategy and prognostic prediction of cognitive dysfunction in patients with MMD.

Choi et al. (2022) conducted a study to explore the relationship between cognitive function and hemodynamics in MMD using hypercapnic challenge blood oxygen level-dependent functional MRI and showed that hemodynamics in the right parietal lobe and white matter correlated with executive function and that hemodynamics in the posterior circulation of the brain correlated with intelligence. This suggests that refined mapping of brain function and structure can be beneficial in improving the ability to predict the changes in cognitive function.

In a cohort of adults with MMD, the authors found that multimodal MRI, including DTI, resting-state functional MRI, could be used to evaluate subtle changes in brain tissue after revascularization (Kazumata et al., 2019). Using resting-state functional MRI, Lei et al. (2017) and Gao et al. (2022) independently found that fluctuations in ALFF and changes in resting-state functional connectivity have the potential to be predictive markers of cognitive function in smoky disease, respectively. In a DTI study, the findings suggested that changes in ADC could reflect changes in cerebral perfusion after revascularization in MMD and thus predict the outcome of cognitive function (Calviere et al., 2020). In another MRI related study, an increase in cerebral microbleeds identified by T2-weighted MRI in patients with MMD might be associated with cognitive dysfunction (Dobashi et al., 2022). In addition, a prospective study showed that revascularization for the target hemisphere also could result in improved cerebrovascular reactivity in the contralateral-non-target hemisphere (Sam et al., 2015).

Positron emission tomography (PET) and single-photon emission computed tomography (SPECT) are widely used in metabolic and hemodynamic studies. In a clinical trial using PET, the authors found that preoperative cerebral oxygen extraction fraction and postoperative cerebral oxygen metabolic rate were strongly associated with cognitive dysfunction in MMD (Hara et al., 2020). In a pediatric MMD cohort, the findings showed that CBF and CVR measured by SPECT might be used to predict cognitive function, and the best prediction period was after the first unilateral revascularization (Nomura et al., 2023). In another study, the results showed that cognitive function in MMD was synchronized with the uptake of Iodine-123-iomazenil by neurons under SPECT (Yoshioka et al., 2024).

One study has shown that intraoperative electrocorticography might be not only sensitive in reflecting and predicting postoperative neurological and cognitive function, but also could be used as a reference index for recipient artery selection in revascularization of MMD (Zhang et al., 2022).

Although the improvement effect of existing treatment programs on cognitive impairment of MMD is still not clear, with the in-depth understanding of the mechanism and characteristics of cognitive impairment of MMD, the treatment and prediction of cognitive function have the opportunity to truly enter clinical practice.

## Future directions and challenges for research on cognitive dysfunction in MMD

The Table 1 summarize the key research in the article. We think that future directions in cognitive dysfunction related to MMD include identification of cognitive impairment characteristics and mechanisms underlying cognitive impairment, early diagnosis of cognitive disorders, management of cognitive dysfunction, and prognostic prediction.

**TABLE 1:** The cognitive research of moyamoya disease.

References	Ethic origin	Patients/ controls	Results/ conclusions
Gatti et al., 2023	United States	83	Children with MMD commonly show cognitive impairment, especially in intelligence and executive function.
Chan et al., 2023	United Kingdom	61	Cognitive dysfunction in adult MMD is common, and the executive function are most vulnerable.
Nakamizo et al., 2022	Japan	20	Cognitive impairment in adult MMD worsens over time.
Shen X. X. et al., 2023	China	115/82	Hypoperfusion can lead to cognitive deficits in non-stroke MMD patients. DSC can be used to assess hemodynamics in MMD
Hara et al., 2019	Japan	31/20	The cognitive impairment in MMD is correlated to the results of PET and NODDI which is used to assess cerebral perfusion.

Recently, research on the mechanisms of cognitive dysfunction in MMD has yielded some results. Xu et al. (2024) have reported that WMHs-induced impairment of the cholinergic pathway was strongly associated with cognitive function in MMD, and the Cholinergic Pathways Hyperintensities Scale has a role in identifying cognitive impairment in MMD. In addition, recent studies have shown that there was impairment in the glymphatic system from the brain in MMD and that the impairment was strongly associated with structural brain changes and cognitive impairment (Zhu et al., 2024; Jin et al., 2024). These findings on the mechanisms of cognitive impairment have the potential to help improve the ability to diagnose and treat cognitive impairment.

Although MMD is rare and heterogeneous, large-scale, long-term, and prospective cohort studies are urgently needed. Higher levels of evidence can favorably guide clinical strategic decision regarding cognitive impairment in MMD.

## Conclusion

In Moyamoya disease, cognitive impairment is common and shows a characteristic pattern of impairment in specific domains, including executive functions, general Intelligence, memory and so on. Neuroimaging findings play a significant role in assessing cognitive impairment and evaluating cognitive impairment and forecasting the prognosis for patients with Moyamoya disease. The effects of revascularization surgery and pharmaceutical interventions on cognitive impairment warrant further investigation. Research on the pathogenesis and etiology associated with Moyamoya disease as well as long-term cohort studies, are important future directions.
